# Natural language processing of biomedical text to map and prioritize protein–disease associations in HFpEF

**DOI:** 10.1016/j.compbiomed.2026.111599

**Published:** 2026-03-03

**Authors:** Clodomir Santana, Chitra Mukherjee, Arnib Quazi, Ronaldo Menezes, Vladimir Filkov, Dibakar Sigdel, Howard Choi, Imo Ebong, Padmini Sirish, Nicholas R. Anderson, Xuan Wang, Heng Ji, JiaWei Han, Baback Roshanravan, Leighton T. Izu, Thomas W. Smith, Nipavan Chiamvimonvat, Colleen E. Clancy, Martin Cadeiras, David A. Liem

**Affiliations:** a Department of Medicine, Division of Cardiovascular Disease, University of California, Davis, USA; b Center for Precision Medicine and Data Science, University of California, Davis, USA; c Department of Computer Science University of Exeter, Exeter, United Kingdom; d AI for Health Center, University of California, Davis, USA; e Department of Medical Informatics, University of California, Davis, USA; f Department of Computer Science, Virginia Polytech Institute and State University, Blacksburg, USA; g Department of Computer Science, University of Illinois, Urbana Champaign, USA; h Department of Nephrology, University of California, Davis, USA; i Department of Pharmacology, University of California, Davis, USA; j Department of Basic Medical Sciences and Translational Cardiovascular Research Center, University of Arizona, College of Medicine, Phoenix, USA; k Department of Physiology and Membrane Biology, University of California, Davis, USA

**Keywords:** Natural language processing, Biomarker discovery, Molecular signatures, Cardiovascular disease

## Abstract

The validation of promising clinical biomarkers, molecular mechanisms, and novel drug targets in cardiovascular disease (CVD) is hindered by a vast and fragmented biomedical literature, which now exceeds 38 million publications indexed in PubMed. To address the central challenge of navigating and synthesizing a huge fragmented biomedical literature base, we applied our validated machine learning–based text-mining algorithm containing natural language processing (NLP) and incorporated this into a **V**al**I**dated **T**ext-mining using **A**dvanced **L**anguage model (**VITAL**) as a complementary framework. Using this approach, we analyzed more than 38 million PubMed abstracts and identified over 5.5 million relevant to six major CVD groups. These curated data then enabled a deep-dive case study on heart failure with preserved ejection fraction (HFpEF). Our computational framework systematically queried, quantified, mapped, and prioritized protein–disease associations, confirming established CVD biomarkers, such as BNP, troponin-I, galectin-3, and renin, and revealing novel protein signatures with potential diagnostic and therapeutic relevance. Ischemic heart disease (IHD, heart attacks), cardiomyopathy (CM, leading to heart failure), and cerebrovascular accidents (CVA, strokes and brain hemorrhages) exhibited the highest protein attribution densities and overlap, suggesting shared molecular pathways. Using HFpEF as a focused case study, our framework identified 5124 proteins associated with this condition, 4879 of which were shared across its major comorbidities (aging, type 2 diabetes/obesity, hypertension, and hyperlipidemia). Additionally, 4991 proteins were co-shared across key pathological mechanisms, including inflammation, mitochondrial dysfunction, and fibrosis, implicating convergent biological networks spanning these domains. To further characterize and prioritize these molecular associations, we performed a series of data science-driven analyses involving HFpEF-associated proteins. The top computationally ranked HFpEF protein candidates were the same top ranked proteins in the comorbidity-domains and in the pathology-domains suggesting that these proteins are important drivers with convergent molecular networks underlying HFpEF. Cross-referencing and validating top-ranked computational HFpEF protein candidates with clinical myocardial and extracardiac biopsy data from HFpEF patients and corresponding controls revealed that most of these proteins are predominantly expressed in the liver, pancreas, adipose tissue, and lymph nodes, rather than in cardiac tissue. This finding supports the emerging concept that HFpEF is fundamentally a multisystemic disorder mediated by inter-organ signaling rather than a disease confined to the heart. Our computational study demonstrates the capacity of text mining to annotate, integrate, and prioritize protein-disease relationships from large-scale textual data, thereby providing a complementary framework to traditional omics approaches for biomarker discovery and drug target identification in CVDs.

## 1. Background and significance

The search for clinical biomarkers, molecular mechanisms, and novel drug targets of disease has traditionally been carried out through biochemical or various omics approaches [1–4]. These studies have led to millions of publications that associated a wide range of multi-omics data (e.g. genes, proteins, and metabolites) to the pathogenesis of specific cardiovascular diseases (CVDs) [5–10]. The challenge is now to discover amongst the accumulated text data across more than 38M publications a set of proteins or metabolites with high priority and expose their hidden molecular patterns that are unique to a specific CVD can serve as disease biomarkers or reveal novel drug targets. To meet this challenge we used our validated machine-learning (ML) empowered text mining system on six main groups of CVDs and other diseases of interest [11–15]. This computational text mining system (Context Aware Semantic Online Analytical Processing, CaseOLAP) uses natural language processing (NLP) approaches that can organize fragmented, heterogeneous textual data and enable a standardized biomedical pattern-recognition (such as hidden molecular signatures in large heterogeneous text data) and extraction of knowledge [11–13,15–20], and incorporated this into a **V**al**I**dated **T**ext-mining using **A**dvanced **L**anguage model (**VITAL**) as a complementary framework.

We applied our text mining algorithm to a textual data corpus of 1,544,691 million publication abstracts, relevant to six main standard CVD groups (based on textbook pathology) identified by their unique Medical Subject Headings (MeSH descriptors) and then focused on heart failure with preserved ejection fraction (HFpEF) as a case study of our interest analyzing comorbidities and pathological mechanisms (totaling over 5.5 million publications all together). HFpEF is a sub-type of HF characterized by a left ventricular ejection fraction (LVEF) ≥50%, multisystemic involvement of extracardiac organs in addition to the heart and a heterogeneous pathophysiologic basis [21–24]. Moreover, in contrast to other types of cardiomyopathies, HFpEF still has limited treatment options [23].

In this computational study we demonstrate that the text mining application not only confirmed clinically established biomarkers (BNP, troponin-I, galectin-3, renin) but also revealed novel protein signatures central to the pathogenesis of the specific CVDs and HFpEF as our use case of interest. Further statistical and ML empowered analyses of these data elucidated the portrayal of each of the six CVDs and HFpEF by the biomedical community publishing on PubMed.

Our study presents a promising computational approach to annotate, analyze and discern biomarkers and protein attributions from vast amounts of biomedical text data. To our knowledge, this study represents the first application of an NLP-based analysis to systematically evaluate protein–HFpEF associations across more than 38 million published studies on the PubMed database rather than focus on text mining electronic health records [25,26] or on bibliometric analysis [27,28].

The ML–based text-mining system incorporating NLP can synergize and guide traditional -omics (bench-work) studies to identify biomarkers by revealing previously unseen protein-to-disease relationships. These novel connections can seed further investigation via clinical and basic translational science studies, and for novel drug target discoveries [21,29–31]. We used HFpEF as a use case of our interest to unveil novel protein-disease relationships and drug targets. Finally, to demonstrate a real-world clinical application and validate our ML–based text-mining system, we cross-referenced our computational findings with a proteomic database of HFpEF patients [32]. In summary, this computational investigation using VITAL presents the first integrated framework to: (1) perform large-scale computational text mining centered on heart failure with preserved ejection fraction (HFpEF), incorporating multi-comorbidity and multi-pathophysiological protein mapping; (2) systematically cross-reference computationally identified proteins with human tissue-specific expression data derived from biopsy specimens; and (3) validate computational predictions using myocardial proteomic profiles from patients with HFpEF.

## 2. Methods

### 2.1. Textual data corpus and protein dataset

**Fig. 1.**
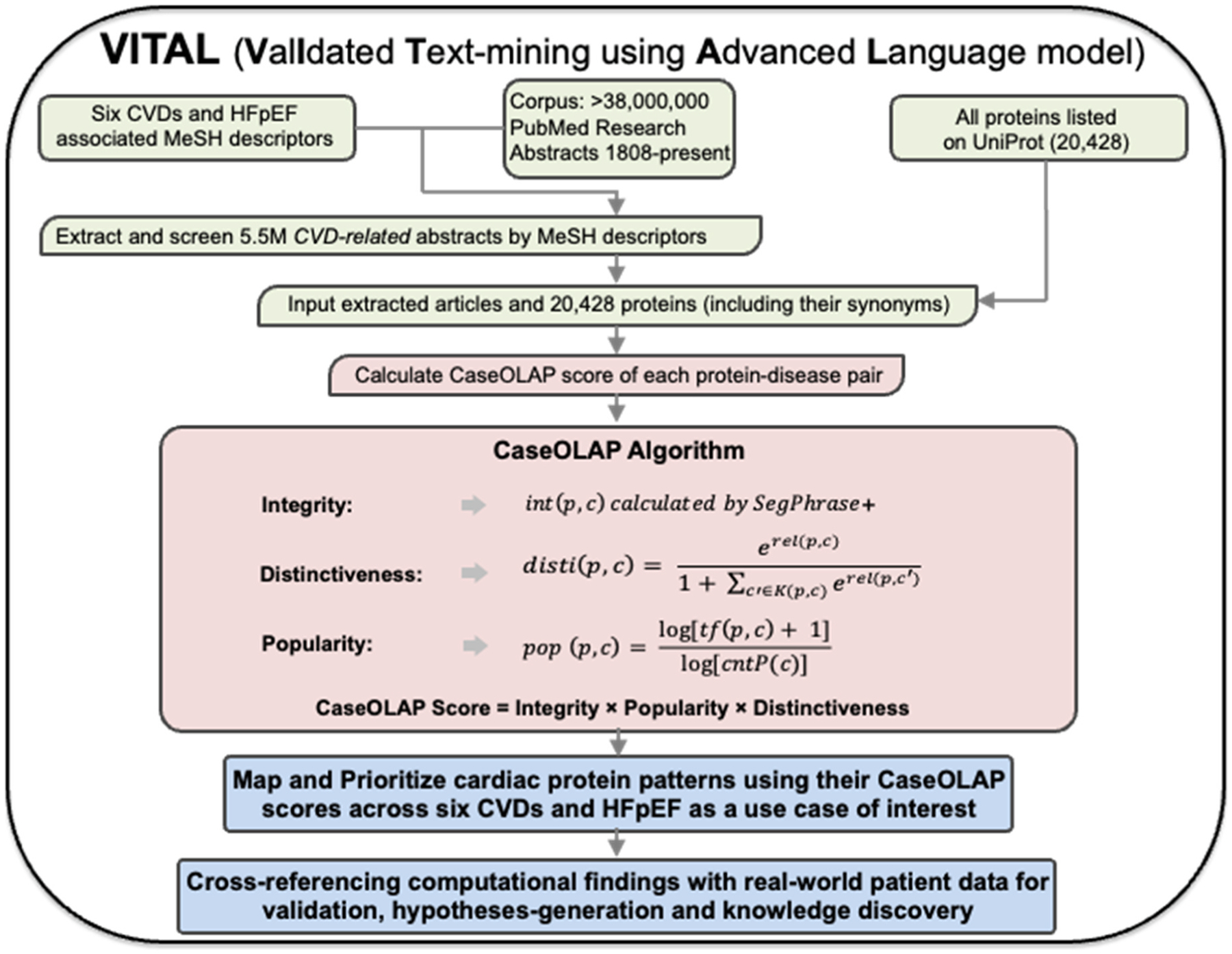
VITAL framework and NLP Algorithm. The framework VITAL (**V**al**I**dated **T**ext-mining using **A**dvanced **L**anguage model) starts with a workflow by screening all research publications available via PubMed to isolate articles associated with our six cardiovascular diseases (CVD) of interest: ischemic heart disease (IHD), cardiomyopathy (CM), cerebrovascular accidents (CVA), valvular heart disease (VD), arrhythmia (ARR), and congenital heart disease (CHD). Employing MeSH descriptors we extracted 1,544,691 CVD-related abstracts. By applying our Text Mining algorithm (CaseOLAP) to our text corpus and 20,428 proteins, we quantified the attribution of each protein to the six CVDs and to our clinical use case of interest, heart failure with preserved ejection fraction (HFpEF). We computed a score for each protein-disease attribution based on three criteria: integrity, popularity, and distinctiveness. The mathematical quantities described in the equations by calculating the distinctiveness and popularity of each protein within the text database are as follows: ‘p’ refers to the phrase, or here, name of the protein; ‘c’ refers to a cell, or one CVD-specific subset of documents from the database of 1,638,533 CVD related abstracts. The term ‘rel(p,c)’ in the distinctiveness equation represents the relevance of the phrase ‘p’ to the cell ‘c’. The term ‘tf(p,c)’ in the popularity equation represents the frequency of phrase ‘p’ in cell ‘c’, and ‘cntP(c)’ is the total frequency of all phrases in cell ‘c’. We analyzed these protein-disease attributions to derive the molecular portrayal.

The full workflow of our study is shown in Fig. 1. We assembled our data set of 5.5M CVD-related article abstracts (including titles and keywords) by using MeSH descriptors to retrieve, out of more than 38M PubMed publications (up till June 2025), all those relevant to the 6 CVDs: cerebrovascular accident (CVA), ischemic heart disease (IHD), cardiomyopathy and heart failure (CM), arrhythmias (ARR), valvular disease (VD), congenital heart disease (CHD) and heart failure with preserved ejection fraction (HFpEF).[12 13] Each CVD, comorbidity, and pathological mechanism was matched to a parent descriptor and its descendent MeSH descriptors on the MeSH descriptor hierarchy and all publications linked to the MeSH descriptor hierarchy (any descriptor from parent MeSH to descendant MeSH) was included into the text corpus of the domain of interest. Only MeSH descriptors, and no key words were used for the building up of the text corpus for each domain of interest. The text corpuses in each domain were built by general MeSH without restriction to CVD context. For the complete list of MeSH descriptors for all CVDs, comorbidities or pathologies see Supplemental Data 1. For our proteins, we employed a dataset of 20,428 proteins available on UniProt. Detailed information acquired from UniProt about these 20,428 proteins (all human proteins available on UniProt) including UniProt ID (uniprot.org), gene symbol, protein name synonyms, and abbreviations can be found in Supplemental Data 2.

### 2.2. Algorithm application and workflow construction

We performed a phrase-mining analysis using the CaseOLAP algorithm (previously developed and validated by our own team) [11–14] to quantify the attributions of 20,428 proteins to each of the 6 CVDs (Fig. 1). Our algorithm integrates three semantic characteristics of publication abstracts to perform its calculations and produce a score: i) *integrity*, ii) *distinctiveness*, and iii) *popularity*. Detailed information about these three characteristics and their calculations are found in Tao et al. [11,33] and Supplemental Data 3.

In brief, *integrity* describes an integral semantic unit that collectively refers to a meaningful concept. In our study, a list of known cardiac protein names was used, each already a semantic unit, was used so that each protein was assigned the maximum *integrity* value of 1.0. *Distinctiveness* measures the exclusivity of a protein’s attribution to a particular CVD, compared to the remaining five. It is calculated using a quantity of relevance, determined by comparing the occurrence of the protein name within the subset of documents pertaining to one CVD against the subsets of documents pertaining to the remaining five CVDs [11 33] (Supplemental Data 3, equation (2)). *Popularity* measures the prevalence of a protein within a specific CVD, depending on how frequently a protein name is mentioned within a subset of documents pertaining to a single CVD (Supplemental Data 3, equation (3)). The product of the three quantities is the final CaseOLAP score for each protein-disease pair with higher scores indicating stronger attributions (Supplemental Data 3, equation (4)). We analyze these protein-to-disease attributions to compute and quantify the molecular portrayal of each CVD group as determined in all relevant publications by the scientific community [12]. Notably, a protein will receive a computational score (between 0 and 1.0) based on the 3 parameters for each domain of interest (HFpEF, aging, T2D/obesity, hypertension, hyperlipidemia, mitochondrial, inflammation, fibrosis). As an example, the top 20 HFpEF proteins refer to the proteins with the highest scores within the domain of HFpEF (later depicted in Figs. 5F and 6E).

### 2.3. Data analysis and bioinformatics

Statistical analyses were performed using the Python programming language (packages: *numpy*, *pandas*, *SciPy, matplotlib)* [34]. We created heatmaps, count plots, a swarm plot, a multiple comparison Venn diagram, temporal analyses, a variety of stacked bar plots using *seaborn, matplotlib* and *pandas* Python packages, and the programming language R.

Hierarchical clustering analysis (Fig. 4B) was performed using complete linkage and the normalized Euclidean distance metric and complete linkage method by the *SciPy* [34] and *seaborn* Python packages. We considered protein scores over CVDs as coordinates and individual proteins as six dimensional vectors (for details see Supplemental Data 3).

### 2.4. Computational infrastructure

All computational analyses were performed on Amazon Web Services (AWS) cloud infrastructure using an EC2 r8g.2xlarge instance (8 vCPUs, 64 GB RAM, ARM-based Graviton3 processors). For data storage, we used AWS S3 buckets totaling 750 GB, which were used to store raw PubMed data, processed corpora, code, and analytical results.

The VITAL framework and all analyses were implemented in Python using open-source libraries. PubMed data retrieval was automated using adapted CaseOLAP Python scripts that interfaced with the NCBI Entrez Programming Utilities (E-utilities) API. For each domain of interest, we created queries using the MeSH descriptor trees (Supplemental Data 1) and implemented batch processing with a rate limit of 10 requests/second to comply with NCBI usage policies. Article metadata (PMID, title, abstract, keywords, publication date, MeSH terms) were retrieved in XML format.

The preprocessing pipeline consisted of sequential steps comprised of (1) PMID deduplication using pandas DataFrame operations, (2) text standardization of protein name synonyms based on UniProt synonyms data (Supplemental Data 2), and (3) domain assignment based on MeSH descriptor annotations.

Protein scores were calculated following the CaseOLAP methodology, which evaluates the proteins in three dimensions: integrity, distinctiveness, and popularity (see Supplemental Data 3, Equation (4)). We leveraged CaseOLAP’s parallel processing capability to efficiently retrieve and process the text corpus.

The code implementing the VITAL framework will be made publicly available on GitHub (https://github.com/chitram1/VITAL/tree/main) upon publication and indexed via Zenodo. Additional technical information is also provided in the supplemental materials.

## 3. Results

We first map 6 CVDs as comparative references and to demonstrate the text mining pipeline, then we focus on HFpEF as a primary use case of interest to illustrate comorbidity, mechanism, and tissue-distribution analysis.

### 3.1. Variable number of proteins attributed to 6 main groups of CVDs

**Fig. 2.**
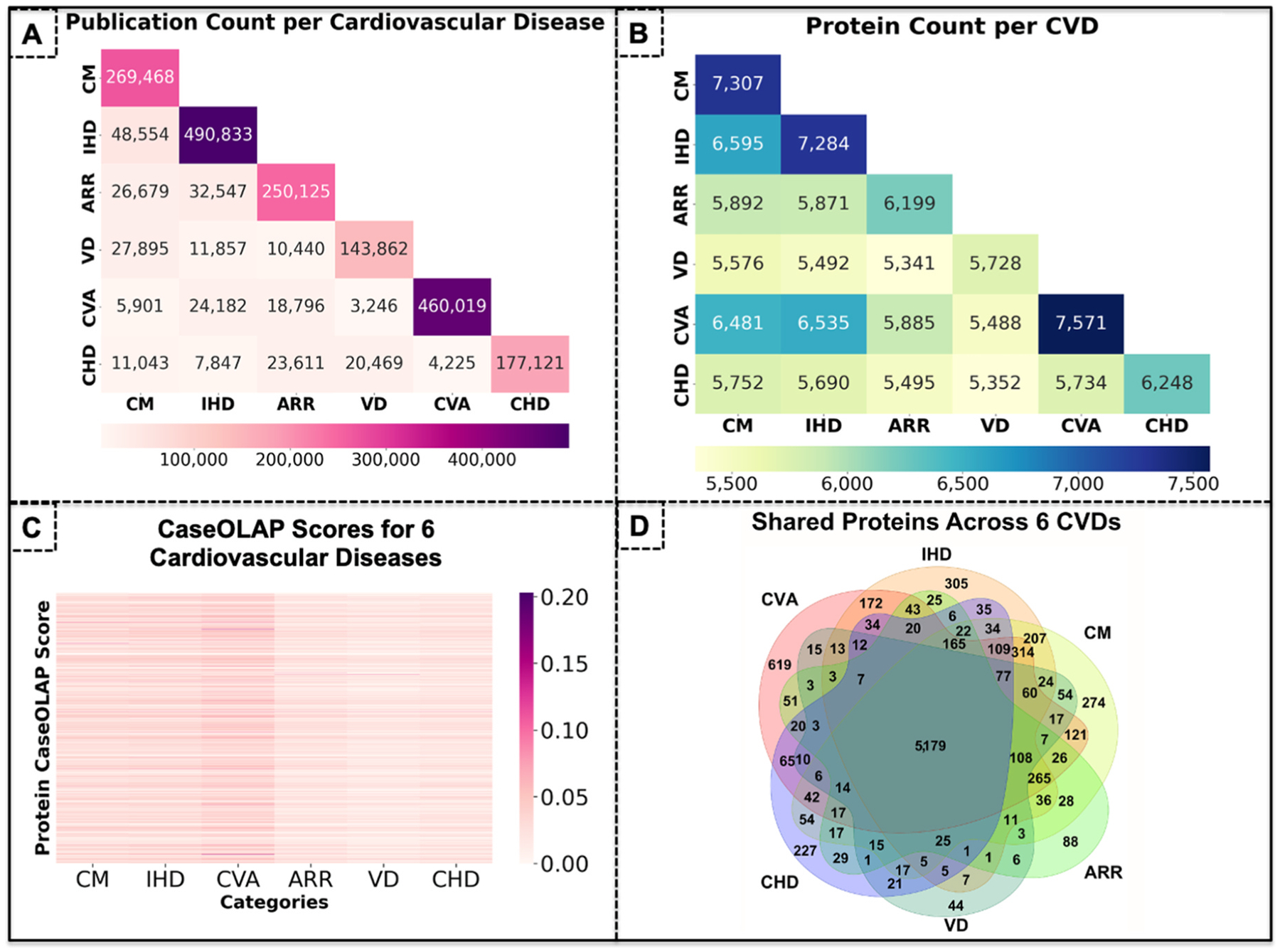
Variable number of cardiac proteins attributed to 6 CVDs. **(A)** We performed a quantitative analysis of the abstracts describing each individual CVD and depicted the results as an abstract count heatmap. The diagonal position highlights that IHD is highly documented with 490,833 abstracts corresponding to 28% of all CVD publications, followed by CVA (460,019; 25% of CVD publications), CM (260,468; 14%), ARR (250,125; 14%), CHD (177,121; 10%), and VD (143,862; 9%). The six CVDs are often studied together and IHD and CM displayed the greatest quantity of shared abstracts (48,554 abstracts). **(B)** To portray the six CVDs by the cardiac proteins of interest, we analyzed the number of proteins attributed to each CVD, creating a protein count heat map. The diagonal position highlights that 7571 proteins have been attributed to CVA; CM and IHD follow closely, with 7307 and 7284 proteins, respectively. IHD, CM, and CVA all share large subsets of protein attributions, while ARR, VD, and CHD share relatively fewer. **(C)** The CaseOLAP algorithm defined protein-disease attributions for over 7400 proteins (from 20,428). Their computational scores are represented as a heat map across the six CVDs. Higher scores, or stronger attributions, are depicted by darker cells. IHD, CM, and CVA exhibit strong patterns of attribution to many proteins, while ARR, CHD, and VD are strongly attributed to relatively fewer proteins. **(D)** We created a multiple comparison Venn diagram to depict proteins attributed to each individual CVD as well as those proteins sharing attributions to all six CVDs. 305 proteins were uniquely ascribed to IHD, 274 proteins to CM, 619 proteins to CVA, 227 proteins to CHD, 88 proteins to ARR, and 44 proteins to VD. 617 proteins out of the 20,428 were attributed to all six CVDs.

The initial ~5.5 million CVD relevant abstracts (from all 38M) were quantified by counting the number of abstracts with MeSH descriptors matching each CVD, and the results are shown as a heatmap (Fig. 2A). IHD is the most studied CVD, having the highest number of corresponding abstracts (490,833 publications), followed by CVA (460,019 publications), CM (269,468 publications), ARR (250,125; publications), CHD (177,121 publications), and VD (143,862 publications). The CVDs are often studied in parallel suggesting shared molecular pathways between CVDs, and many abstracts are labeled with MeSH descriptors corresponding to multiple CVDs. In particular, IHD and CM displayed the greatest quantity of shared abstracts (48,554 publications), followed by IHD and ARR (32,547 publications), CM and VD (27,895 publications), and IHD and CVA (24,182 publications).

In the protein count map (Fig. 2B), CVA displayed the highest number of attributed proteins (7,571), followed closely by CM (7,307) and IHD (7,284). These three CVDs also exhibited the highest numbers of shared proteins: the most were shared between CM and IHD (6,595), and second most between IHD and ARR (5,871), suggesting overlapping molecular pathways. CHD (6,248), ARR (6,199), and VD (5,728) exhibited relatively fewer protein attributions.

Our Text Mining algorithm defined protein-to-disease attributions for over 9198 of the 20,428 proteins. The heatmap in Fig. 2C shows that IHD, CM, and CVA exhibit strong patterns of attribution (more darker cells indicating a higher CaseOLAP score) to more proteins, compared to ARR, CHD, and VD with quantitatively fewer proteins showing strong patterns of attribution. As depicted in a Venn diagram, we analyzed the number of proteins exclusively attributed to each individual CVD as well as the number of proteins that share attributions to two or more CVDs (Fig. 2D). Hence, CVA exhibited the highest number of proteins uniquely associated with this disease (619 proteins), followed by IHD (305), CVA (352), CM (274), CHD (227), ARR (88) and VD (44). From the dataset of 20,428 heart related proteins, only 6133 were attributed to all six CVDs.

### 3.2. Distribution of the number of proteins across their disease association to 6 CVDs

**Fig. 3.**
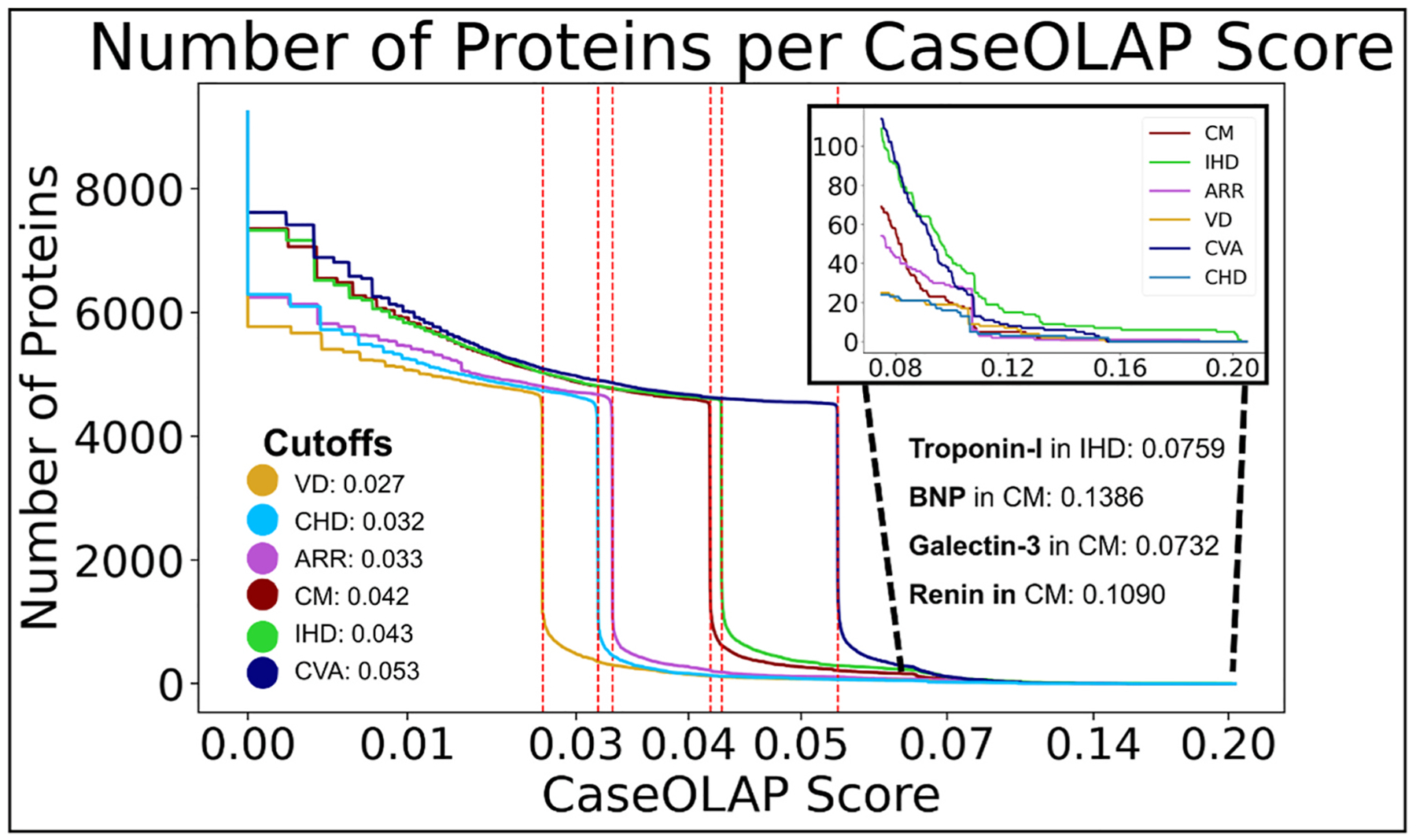
Distribution of the number of proteins across their associations to 6 CVDs. **(A)** The distribution of protein counts (Y-axis) across the CaseOLAP protein-disease scores (x-axis) for the 6 CVDs (colored lines) is displayed. Most proteins retrieved computational scores <0.03 for each CVD, indicating proteins loosely attributed to the 6 CVDs. Proteins with higher scores are defined as displaying moderate attributions. We observed for each of the 6 CVDs that they displayed a cut-off value of the computational CaseOLAP score. Hence, in each CVD beyond its cut-off score, the number of proteins drastically decreased and proteins with a score higher than the cut-off would be more exclusive to its CVD (with little or no overlap with the other CVDs). Known biomarkers, such as troponin-I, BNP, renin, and galectin-3, all retrieved scores corresponding to strong protein-disease attributions with a computational score higher than the cut-off value in the CVD in which the protein is a known clinical biomarker.

To analyze how the number of proteins is distributed over the CaseOLAP scores reflecting the protein-to-disease spectrum, we displayed the protein counts (y-axis) across the CaseOLAP score (x-axis). Each CVD is depicted as a different color (Fig. 3A). Notably, the majority of the 20,428 proteins did not receive a Text Mining score indicating no correlation to any CVD group. A total of 9198 proteins received at least one score in a CVD. We observed within each of the 6 CVDs that the higher their protein CaseOLAP score, the more exclusive the protein is attributed to the CVD. We also observed that the CaseOLAP scores exhibited a cut-off value within each CVD (Fig. 3A). The cut-off value for each CVD was not chosen arbitrarily but determined based on the CaseOLAP score, where the number of proteins on the y-axis declines steeply with each increase in x. This value was statistically derived by finding the largest negative first derivative of the CaseOLAP score on the x-axis, for which the number of proteins on the y-axis shows the steepest drop. In Fig. 3A the cut-off values are depicted for each CVD as the CaseOLAP score where the number of proteins steeply drop. Proteins with a higher score beyond the cut-off value have high exclusiveness for the CVD. Accordingly, gold standard clinical biomarkers, such as troponin-I (indicator of myocardial injury in IHD), brain natriuretic peptide (BNP, indicator of myocardial stretch or stress in CM), angiotensin converting enzyme (ACE, regulating hemodynamics in heart failure), renin (regulator of hemodynamics), tumor necrosis factor (TNF, a cytokine increased in inflammatory reactions, especially during cardiac tissue damage) and galectin-3 (a pro-inflammatory protein highly relevant in CM and Heart Failure) all received CaseOLAP scores beyond the cut-off value of exclusiveness in their respective disease, indicating strong protein-to-disease relationships. Accordingly, we observed a cutoff value to define levels of relationship serving to highlight the uniqueness, as well as the relevance, or lack thereof, of the proteins to the six CVDs. Proteins with scores greater than their cutoff values (as depicted for each CVD in Fig. 3) were defined as displaying strong attribution to a single CVD. Proteins with scores below their cutoff values were defined as moderately attributed to one (or more) CVD(s). Most proteins received scores below the cutoff values for each CVD and were defined as loosely attributed to the six CVDs. The majority of proteins exhibited loose association to two or more CVDs, while relatively few proteins exhibited strong associations to two or more CVDs. Proteins exhibiting at least one score greater than the cutoff value in a CVD were largely unique to that CVD. We observed that proteins rarely exhibit high computational scores beyond a cutoff value in more than one disease, and as such, they may represent exclusive molecular signatures, and potential clinical biomarkers with diagnostic and prognostic relevance for their respective CVD group, or even potential new drug targets.

### 3.3. Querying and mapping all reported proteins in HFpEF using an NLP approach

**Fig. 4.**
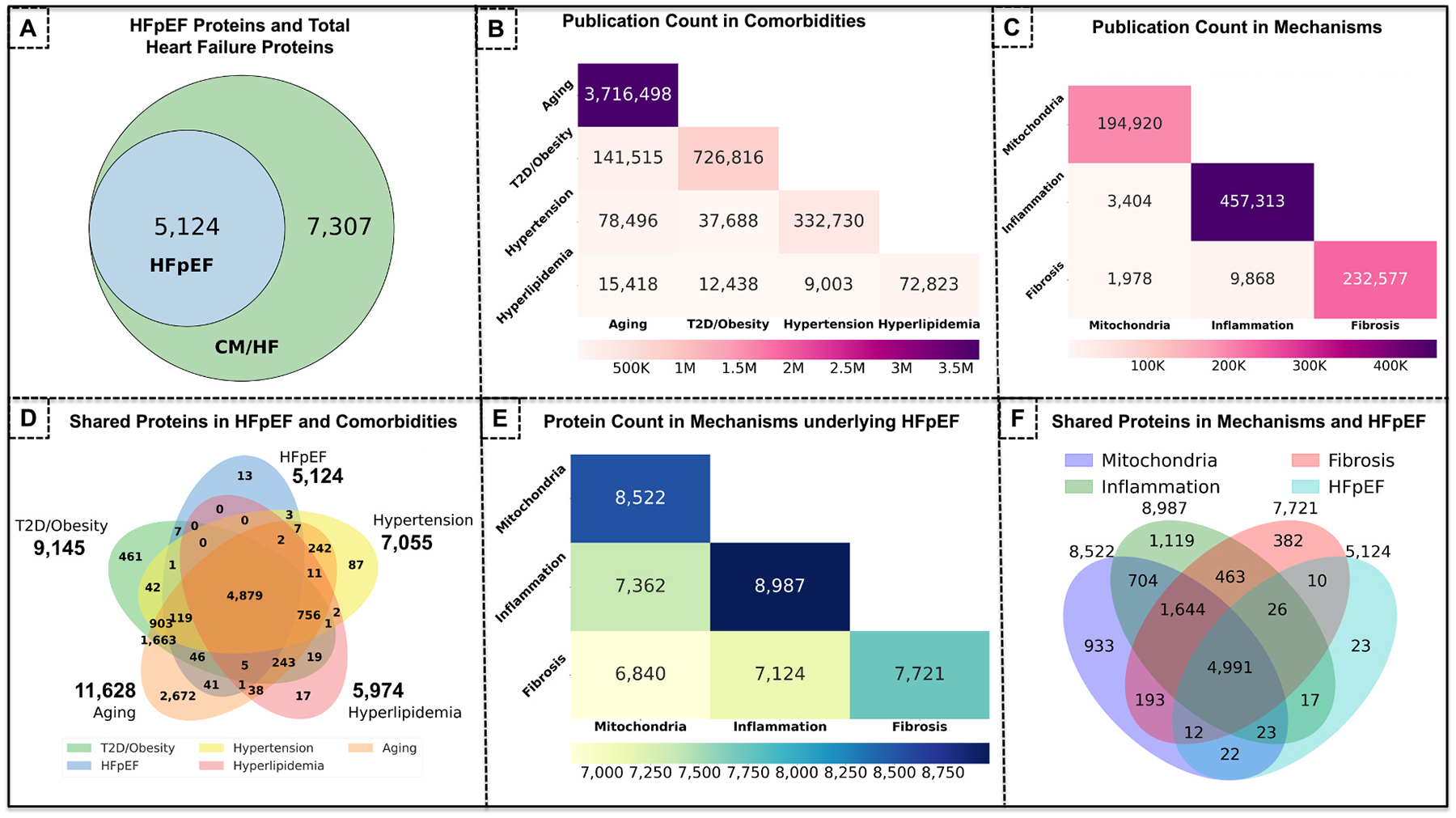
Querying and Mapping all Reported HFpEF Proteins Using an NLP Approach. Well-recognized underlying pathophysiological mechanisms in HFpEF are Mitochondrial function, Inflammation, and Fibrosis each playing a relevant role in diastolic dysfunction of the heart. **(A)** we queried 5124 proteins described in HFpEF publications out of a total of 7307 proteins described in any type of heart failure publications on PubMed. **(B)**, we allocated all publications on PubMed addressing the HFpEF comorbidities with Aging exhibiting the highest number (3,716,498 publications), followed by T2D and obesity (combined as T2D/Obesity) with 726,816 publications, hypertension with 332,750 publications, and hyperlipidemia with 72,823 publications. T2D/obesity shared the most overlapping publications with aging (141,515) followed by hypertension (37,688), and hyperlipidemia (9,003). **(C)** We allocated all publications on PubMed addressing well-recognized HFpEF pathologies with inflammation exhibiting the highest publication count (457,313) followed by fibrosis (232,577) and mitochondrial function (194,920). Inflammation also shared the most overlapping publications with fibrosis showing many common elements between these pathologies. **(D)** We present an overview of all queried proteins allocated across HFpEF (including a total of 5124 proteins), T2D/obesity (including a total of 9145 proteins), aging (a total of 11,628 proteins), hyperlipidemia (a total of 5974 proteins), and hypertension (a total of 7055 proteins). A total of 4879 overlapped in all domains including HFpEF. We believe that these 4879 proteins that are shared in all 5 domains may be involved in biological pathways and networks that are important in the pathophysiology of HFpEF and its comorbidities (aging, T2D/obesity, hyperlipidemia and hypertension). **(E)** We allocated all proteins described in prominent underlying mechanisms of HFpEF showing the highest number of proteins in inflammation (8987 proteins), followed by mitochondrial function (8522 proteins), and fibrosis (7721 proteins). **(F)** We presented all queried HFpEF proteins and their overlap across the domains of interest: mitochondrial function, inflammation, and fibrosis. Out of a total of 10,552 proteins involved in at least one of the domains of interest (i.e., HFpEF, mitochondria, inflammation, fibrosis) a subset of 4991 proteins are shared in all 4 domains.

**Fig. 5.**
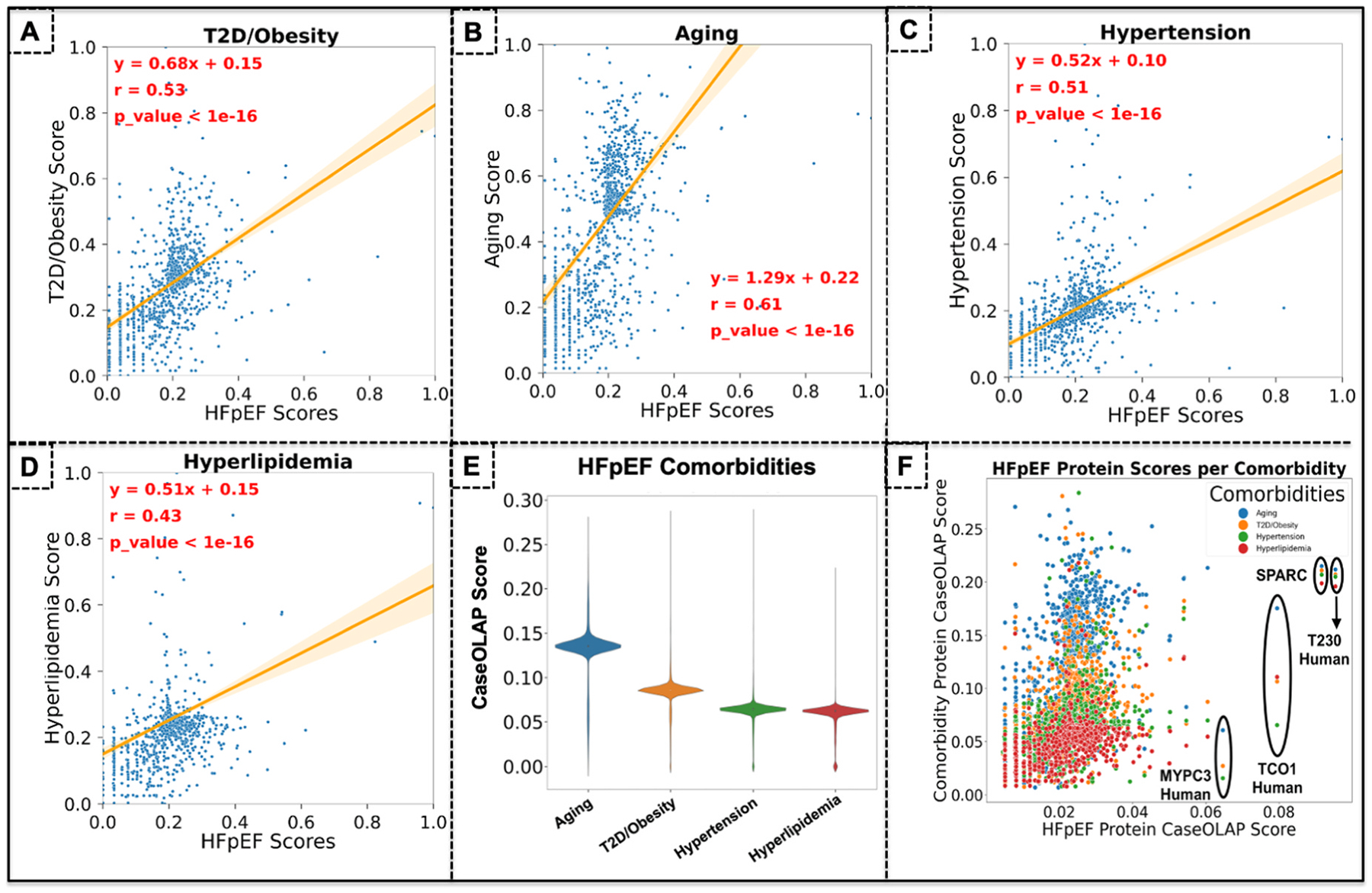
Mapping and Prioritizing Protein-Disease Associations in HFpEF across Comorbidities. We analyzed the protein-disease associations across HFpEF and its comorbidities. **(A)** We conducted a regression analysis of all HFpEF proteins co-shared with T2D/obesity. Hence, the CaseOLAP protein scores for T2D/obesity are on the Y-axis and the scores for HFpEF on the X-axis. Linear regression of the protein scores across HFpEF and T2D/obesity showed y = 0.68x + 0.15 (P ≤ 0.05) with an r value of 0.53. **(B)** A linear regression analysis of all HFpEF proteins co-shared with aging showed y = 1.20x + 0.22 (P ≤ 0.05) with a higher r value of 0.61 compared to T2D/obesity. **(C)** Linear regression analysis of HFpEF proteins and hypertension showed y = 0.52x + 0.10 (P ≤ 0.05) exhibiting a similar r value as T2D/obesity (r = 0.51). **(D)** Hyperlipidemia showed a lower linear regression y0.51x + 0.15 with the lowest r value of 0.43. Although all 4 comorbidities are established high risk factors aging included more proteins that have higher computational scores in both HFpEF and aging as reflected in the highest r value. **(E)** The CaseOLAP protein scores are presented for all four comorbidities in a violin plot to analyze their distribution, outliers, and mean values. All comorbidities showed similar values in outliers of their protein scores between 0 and 0.28. The mean value of all protein scores was higher in aging (0.14), followed by T2D/obesity (0.09). Both hypertension and hyperlipidemia had lower mean values, respectively (0.07) and (0.07). All comorbidities showed a normal distribution of their computational protein scores. **(F)** we present a scatter plot with the CasOLAP score for all HFpEF proteins in the X-axis and their respective scores for the comorbidities on the Y-axis (scores for aging in blue, T2D/obesity in orange, hypertension in green, hyperlipidemia in red).

**Fig. 6.**
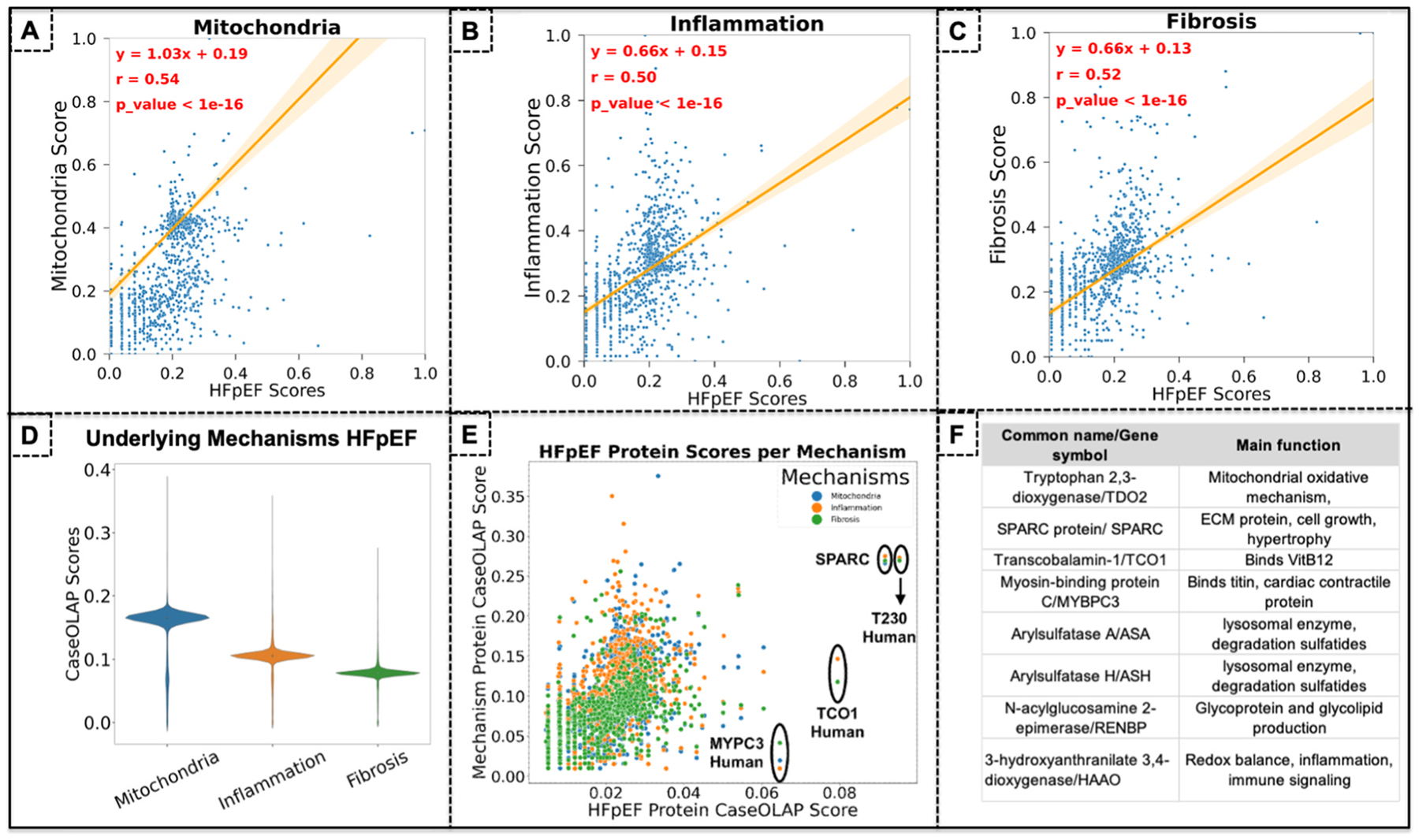
Mapping and Prioritizing Protein-Disease Associations in HFpEF across Pathological Mechanisms. We next analyzed the protein-disease associations across HFpEF and its pathological mechanisms. **(A)** Linear regression of the protein scores across HFpEF and mitochondria showed y = 1.03x + 0.19 (P ≤ 0.05) with an r value of 0.54. **(B)** Linear regression of the protein scores across HFpEF and inflammation showed y = 0.66x + 0.15 (P ≤ 0.05) with an r value of 0.50. **(C)** Linear regression of the protein scores across HFpEF and fibrosis showed y = 0.66x + 0.13 (P ≤ 0.05) with a r value of 0.52. The higher r value in mitochondria compared to inflammation and fibrosis indicates that it includes more HFpEF proteins that also have a higher computational protein score for mitochondria as compared to the other pathological mechanisms. **(D)** The violin plot for the three pathological mechanisms showed the largest outliers within mitochondria between 0 and 0.4 and the smallest range for fibrosis between 0 and 0.28. The mean value of all protein scores was significantly higher in mitochondria (0.18), followed by inflammation (0.1) and fibrosis (0.08). All comorbidities showed a normal distribution of their computational protein scores. **(E)** We present a scatter plot with all HFpEF proteins scores in the X-axis and their respective scores for the pathological mechanisms on the Y-axis (scores for mitochondria in blue, inflammation in orange, fibrosis in green). The two highest ranking HFpEF proteins also had simultaneous high scores for all three pathological mechanisms, indicating that these two proteins are concurrently important in HFpEF, it’s comorbidities, and the three pathological mechanisms.

We aimed to analyze protein-to-disease attributions in heart failure with preserved ejection fraction (HFpEF) as our clinical domain of interest. Notably, HFpEF is a subtype of heart failure strongly correlated to comorbidities such Type 2 Diabetes (T2D), obesity, hypertension, hyperlipidemia and aging (the risk for HFpEF is much increased at higher age) [21,26,35]. Well-recognized underlying pathophysiological mechanisms in HFpEF are mitochondrial function, inflammation, and fibrosis each playing a relevant role in diastolic dysfunction of the heart [21,36]. As depicted in Fig. 4A, we queried 5124 proteins described in HFpEF relevant publications out of a total of 7307 proteins described in any type of heart failure publications on PubMed. If a protein was mentioned in only one HFpEF publication, we still included this in our query. In Fig. 4B, we cross-allocated all PubMed publications addressing the HFpEF comorbidities with aging exhibiting the highest number (3, 716,498 publications), followed by T2D and obesity (combined as T2D/obesity) with 726,816 publications, hypertension with 332,750 publications, and hyperlipidemia with 72,823 publications. This indicates that out of HFpEF comorbidities aging has the utmost research popularity as reflected in the high number of publications followed by T2D/obesity, hypertension, and hyperlipidemia. We also observed that T2D/obesity and aging co-occurred in the most publications (141,515) followed by hypertension (37,688), and hyperlipidemia (9,003) indicating that T2D/obesity may have non-trivial overlap with aging and the two are more correlated. In addition to comorbidities, we wanted to map all publications and proteins-to-disease attributions in established pathophysiological mechanisms of HFpEF. As depicted in Fig. 4C, we allocated all publications on PubMed addressing well-recognized HFpEF pathologies with Inflammation exhibiting the highest publication count (457,313) followed by fibrosis (232,577) and mitochondrial function (194,920). As inflammation is involved in many diseases beyond the heart, it is expected to exhibit the highest number of publications followed by mitochondria and fibrosis. inflammation also shared the most co-occurences publications with fibrosis showing many common elements between these pathologies. In Fig. 4D we present an overview of all queried proteins allocated across HFpEF (including a total of 5124 proteins), T2D/obesity (including a total of 9145 proteins), aging (a total of 11,628 proteins), hyperlipidemia (a total of 5974 proteins), and hypertension (a total of 7055 proteins). A total of 12,281 proteins were involved across all 5 domains of interest of which 4879 overlapped in all domains including HFpEF. We believe that these 4879 proteins that are shared in all 5 domains may be involved in biological pathways and networks that are important in the pathophysiology of HFpEF and its comorbidities (aging, T2D/obesity, hyperlipidemia and hypertension) and we conducted additional analyses on these proteins described later (Fig. 5F). We observed that aging shared the most proteins with T2D/obesity (1666 proteins) suggesting that these 2 comorbidities share the most common biological pathways involving proteins among these 5 domains of interest. In Fig. 4E we allocated all proteins described in prominent underlying mechanisms of HFpEF showing the highest number of proteins in Inflammation (8987 proteins), followed by mitochondrial function (8522 proteins), and fibrosis (7721 proteins). Although the number of publications is very different in-between the underlying mechanisms (Fig. 4C) the total number of proteins within each domain of interest still fall in the same range. Hence, while inflammation has twice as many publications compared to mitochondria and fibrosis, it appears that many published proteins are often repeated (republished) across publications on inflammation. The Venn diagram in Fig. 4F presents all queried and allocated proteins in HFpEF, mitochondrial function, inflammation, and fibrosis as well as their overlap across the domains of interest. Out of a total of 10,552 proteins involved in at least one of the domains of interest (i.e., HFpEF, mitochondria, inflammation, fibrosis) a subset of 4991 proteins are shared in all 4 domains. These 4991 proteins that are shared in all 4 domains of interest may be involved in biological pathways and networks that are important in the pathological mechanisms of HFpEF (mitochondria, inflammation, fibrosis) and we conducted additional analyses on these proteins described later (Fig. 6C).

### 3.4. Ranking and prioritizing protein-disease associations in HFpEF across its comorbidities

We analyzed the protein-disease associations in our comorbidities of interest across HFpEF proteins. The idea behind the correlation analysis presented in Fig. 5A–D is to assess whether CaseOLAP scores appropriately captured known relationships between HFpEF and its comorbidities. For each comorbidity, we identified proteins associated with both HFpEF and the comorbidity, then correlated their respective CaseOLAP scores across both conditions. We expected to find moderate positive correlations, indicating that proteins relevant to HFpEF would also score highly for related comorbidities. In Fig. 5A we present a scatterplot with a regression analysis of all HFpEF proteins co-shared with T2D/obesity (a total of 5057 shared proteins as depicted in the scatterplot), with the CaseOLAP protein scores for T2D/obesity on the Y-axis and the scores for HFpEF on the X-axis. Linear regression of the protein scores across HFpEF and T2D/obesity showed a trend of y = 0.68x + 0.15 (p ≤ 0.05) with an r value of 0.53. In Fig. 5B we present a scatter plot with a regression analysis of all HFpEF proteins co-shared with aging (a total of 5099 proteins). Linear regression of the protein scores across HFpEF and aging showed a trend of y = 1.20x + 0.22 (P ≤ 0.05) with a higher r value of 0.61. The higher r value in aging compared to T2D/Obesity indicates that it includes more HFpEF proteins that also have a higher computational protein score for Aging. In Fig. 5C a regression analysis of HFpEF proteins and hypertension showed a trend of y = 0.52x + 0.10 (P ≤ 0.05) showing a similar r value as T2D/obesity (r = 0.51). hyperlipidemia showed a lower linear regression trend of y0.51x + 0.15 with the lowest r value of 0.43. Although all 4 comorbidities are established high risk factors that are strongly correlated to HFpEF we observed that aging included more proteins that have higher computational scores in both HFpEF and aging as reflected in the highest r value (Fig. 5B). This signifies that many proteins that have an important role in HFpEF (reflected in higher CaseOLAP scores) also have an important role in aging. Hypertension and T2D/obesity almost had identical r values with a similar number of high scoring proteins that also have a high score in HFpEF. Although Hyperlipidemia is an established comorbidity in heart disease, it showed the lowest r value compared to the other 3 comorbidities including less proteins with a high CaseOLAP score in both HFpEF and hyperlipidemia. Our results implies that aging may share more biological pathways with HFpEF compared to the other comorbidities. The observed correlations (r = 0.43–0.61) suggest biological overlap between HFpEF and these comorbidities. However, due to disease-specific mechanisms, stronger correlations were not expected. Each comorbidity has unique etiological pathways involving proteins less relevant to HFpEF pathophysiology.

In Fig. 5E the CaseOLAP protein scores are presented for all four comorbidities in a violin plot to analyze their distribution, outliers, and mean values. All comorbidities showed similar values in outliers of their protein scores between 0 and 0.28. The mean value of all protein scores was higher in Aging (0.14), followed by T2D/Obesity (0.09). Both Hypertension and Hyperlipidemia had lower mean values, respectively (0.07) and (0.07). All comorbidities showed a normal distribution of their computational protein scores. In Fig. 5F we present a scatter plot with the CasOLAP score for all HFpEF proteins in the X-axis and their respective scores for the comorbidities on the Y-axis (scores for aging in blue, T2D/obesity in orange, hypertension in green, hyperlipidemia in red). In Fig. 5F on the X-axis we can appreciate the top 5 proteins with the highest computational scores for HFpEF (indicating a high popularity and distinctiveness in HFpEF). Accordingly, the highest ranked protein was tryptophan 2,3-dioxygenase (with UniProt entry name T230_HUMAN and Gene symbol TDO2) which simultaneously also showed the highest scores >0.20 for all 4 comorbidities. This indicates an important role for tryptophan 2,3-dioxygenase and its biological molecular pathways in HFpEF, aging, T2D/obesity, hypertension and hyperlipidemia. Interestingly, TDO2 is an important metabolite in biological pathways of the microbiome and central in many disease mechanisms such as in heart failure [37], Alzheimer [38], the immune system [39], and chronic stress [40]. The second top ranked HFpEF protein was Basement Membrane Protein 40 (with UniProt gene name SPARC) which is a extracellular matrix protein and an important key player in cardiac remodeling such as ventricular hypertrophy and dilation [41–43]. As SPARC is simultaneously top ranked in aging, T2D/obesity, hypertension and hyperlipidemia it suggests that these comorbidities of HFpEF may be linked in cardiac remodeling and diastolic dysfunction. The third ranked HFpEF protein is transcobalamin-1 (with UniProt gene name TCO1) which is an important protein in the respiratory transport chain of the mitochondrial inner membrane during energy production in the cell. TCO1 had simultaneous high scores in aging, T2D/obesity and hyperlipidemia and exhibited a moderate score for hypertension. TCO1 is often linked in biological processes underlying oxidative stress. The fourth high ranked HFpEF protein was Myosin-binding protein C (UniProt gene name MYPC3) which is a cardiac contractile protein important is cardiac function, contractility and remodeling. While MYPC3 is the fourth ranked HFpEF protein, it had lower scores for aging, T2D/obesity and hypertension (all <0.06) and was not found in hyperlipidemia (no CaseOLAP score). The fifth highest ranked HFpEF protein was Arylsulfatase A (gene name ASA). ASA had simultaneous moderate to low scores in T2D/obesity, hypertension and hyperlipidemia (<0.05) but scored high in aging (0.21). This suggest that ASA may be an important protein with biological pathways in aging and HFpEF.

### 3.5. Ranking and prioritizing protein-disease associations in HFpEF across pathological mechanisms

Like the comorbidities, we next analyzed the protein-disease associations in our pathological mechanisms of interest across HFpEF. In Fig. 6A we present a scatterplot with a regression analysis of all HFpEF proteins co-shared with mitochondria (a total of 5048 shared proteins as depicted in the scatterplot), with the CaseOLAP protein scores for mitochondria on the Y-axis and the scores for HFpEF on the X-axis. Linear regression of the protein scores across HFpEF and mitochondria showed a trend of y = 1.03x + 0.19 (P ≤ 0.05) with an r value of 0.54. In Fig. 6B we present a scatter plot with a regression analysis of all HFpEF proteins co-shared with inflammation (a total of 5057 proteins). Linear regression of the protein scores across HFpEF and inflammation showed a trend of y = 0.66x + 0.15 (P ≤ 0.05) with a higher r value of 0.50. Similarly, Fig. 6C present a regression analysis of HFpEF proteins co-shared with fibrosis (5027 proteins). Linear regression of the protein scores across HFpEF and fibrosis showed a trend of y = 0.66x + 0.13 (P ≤ 0.05) with a r value of 0.52. The higher r value in mitochondria compared to inflammation and fibrosis indicates that it includes more HFpEF proteins that also have a higher computational protein score for mitochondria as compared to the other pathological mechanisms. Similar to the comorbidity analysis, the observed moderate correlations suggest biological overlap between HFpEF and these underlying mechanisms while preserving pathway-specific patterns. Given that each mechanism involves proteins with specialized functions that may be less central to HFpEF pathophysiology, stronger correlations could indicate potential biases in the text corpus. In this case, papers mentioning HFpEF and a given mechanism at the same time might artificially inflate the correlations and CaseOLAP scores. In Fig. 6D the violin plot for the three pathological mechanisms showed the largest outliers within mitochondria between 0 and 0.4 and the smallest range for fibrosis between 0 and 0.28. The mean value of all protein scores was higher in mitochondria (0.18), followed by inflammation (0.1) and fibrosis (0.08). All comorbidities showed a normal distribution of their computational protein scores. In Fig. 6E we present a scatter plot with the CasOLAP score for all HFpEF proteins in the X-axis and their respective scores for the pathological mechanisms on the Y-axis (scores for mitochondria in blue, inflammation in orange, fibrosis in green). While the overall corpus of HFpEF proteins overlapping with mitochondria, inflammation, and fibrosis are different from the protein corpus in comorbidities (Fig. 5F), the top HFpEF proteins in the overlap of pathological mechanisms were similar to the proteins in the overlap of comorbidities namely, TDO2, SPARC, TCO1, MYPC3, and ASA. Interestingly, the two highest ranking HFpEF proteins also had simultaneous high scores for all three pathological mechanisms, potentially indicating that these two proteins are concurrently important in HFpEF, it’s comorbidities, and the three pathological mechanisms.

### 3.6. Cross-referencing and validating high-ranked computationally acquired HFpEF proteins with myocardial biopsies from HFpEF patients

To validate high ranked proteins identified through our computational NLP approaches, we employed a myocardial tissue proteomics dataset derived from patients with HFpEF [32]. Endomyocardial tissue specimens were obtained from the right ventricular septum of HFpEF patients using standard clinical biopsy procurements alongside age-matched control tissue from donor hearts (n = 9 controls, n = 10 HFpEF). This dataset comprised a total of 2180 proteins. It is important to note that the HFpEF cardiac biopsy cohort used for cross-referencing is limited to a small sample size (n = 10), and therefore the corresponding findings should be interpreted with caution and not be over-generalized. Nevertheless, protein abundance patterns were highly consistent across HFpEF samples and demonstrated a clear contrast relative to control biopsies, supporting the internal consistency of the dataset.

**Fig. 7.**
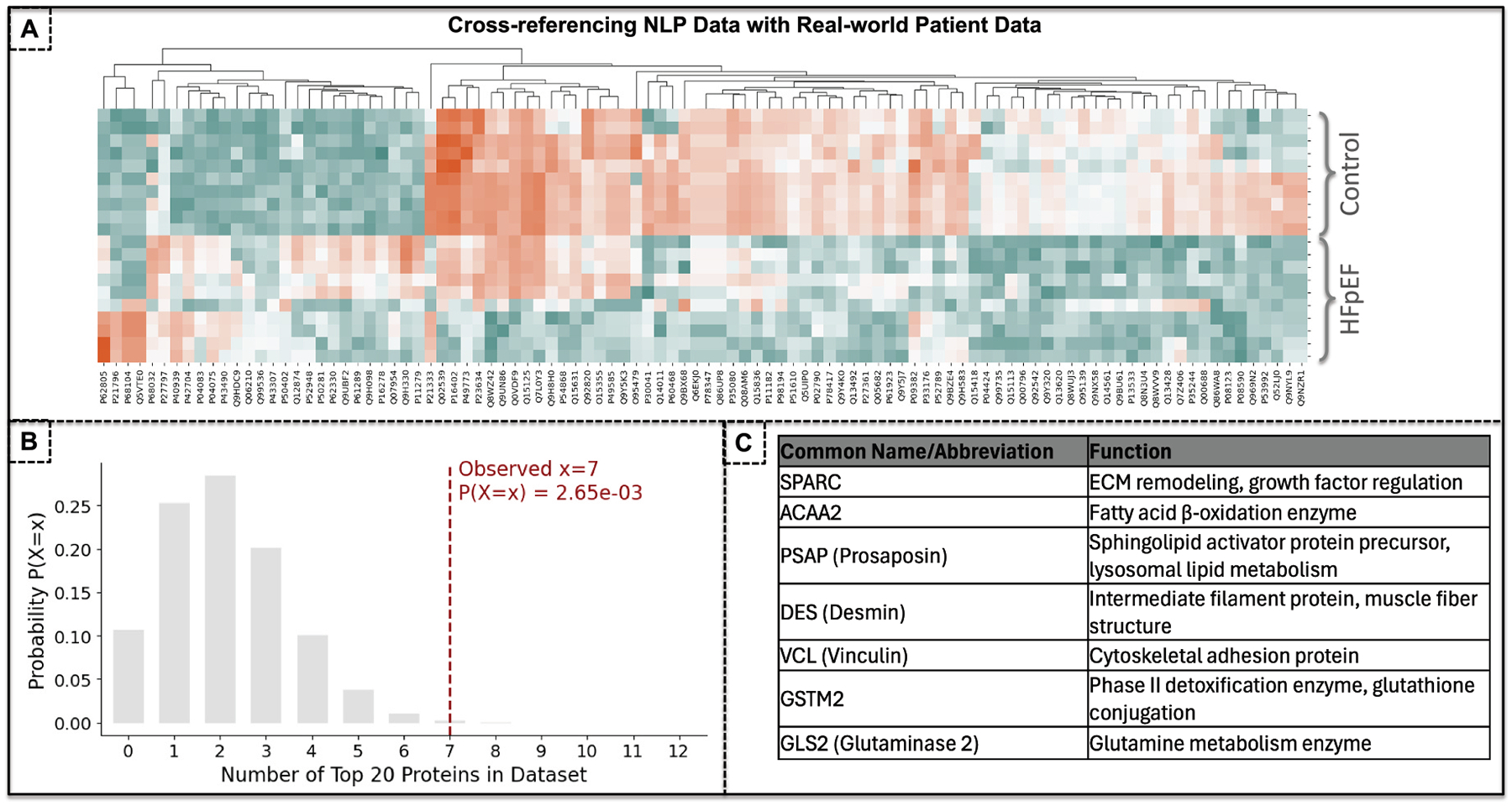
Cross-referencing and validating top-ranked computationally derived HFpEF-associated proteins using myocardial biopsies from patients. To validate high ranked proteins identified through our computational NLP approaches, we employed a myocardial tissue proteomics dataset derived from HFpEF patients and healthy controls [32]. The proteins were sorted by mean difference (descending) and p-value (ascending). **(A)** shows the top 100 proteins most differentially expressed between myocardial biopsies from HFpEF patients, and controls, ranked by mean difference and statistical significance. We ranked the top 20 CaseOLAP proteins for HFpEF and found that seven overlapped with the top 100 differential proteins from myocardial patient biopsies. We assessed the statistical relevance and significance of these findings by leveraging a hypergeometric test to evaluate whether a set of observed “hits” (e.g., overlapping disease-relevant proteins) is enriched beyond what would be expected by random chance. **(B)** The bar plot compares the expected number of disease-relevant proteins (based on chance alone) to the actual number observed. The low p-value of 0.00265 is statistically significant, indicating that the observed overlap is unlikely to have occurred randomly and suggesting biological relevance or enrichment. We found an enrichment factor of 2.65, meaning that the size of the overlap set found is 2.65 times higher than what would be expected by chance. **(C)** The overlapping proteins that were identified using our NLP algorithm are presented highlighting vinculin, desmin, and SPARC. The results suggest that computationally identified high-ranked protein-disease associations are reflected in real world HFpEF patient samples.

To identify differential proteins between the HFpEF and control groups, we compared protein expression levels between the two groups. For each protein, we performed a Kolmogorov-Smirnov (KS) [44] test to assess whether the distributions of expression values were significantly different between HFpEF and control. We also calculated the mean expression for each group and the absolute difference between their mean values. Finally, the proteins were sorted by mean difference (descending) and p-value (ascending). Fig. 7A shows the top 100 proteins most differentially expressed between myocardial biopsies from HFpEF patients and controls, ranked by mean difference and statistical significance. Next, we ranked the top 20 CaseOLAP proteins for HFpEF and found that seven overlapped with the proteins from myocardial patient biopsies. We assessed the statistical relevance and significance of these findings by leveraging a hypergeometric test [45] to evaluate whether a set of observed “hits” (e.g., overlapping disease-relevant proteins) is enriched beyond what would be expected by random chance. Out of a total background of 20,428 human proteins (all proteins on UniProt), 2180 proteins were present in the myocardial patient samples. Among these, 7 (k) were found to belong to the CaseOLAP list of 20 disease-relevant proteins (K). The bar plot in Fig. 7B compares the expected number of disease-relevant proteins (based on chance alone) to the actual number observed. The low p-value of 0.00265 is statistically significant, indicating that the observed overlap is unlikely to have occurred by chance and suggesting biological relevance or enrichment. In this case, we found an enrichment factor of 2.65, meaning that the size of the overlap set found is 2.65 times higher than what would be expected by chance. Accordingly, our natural language processing analyses of the PubMed corpus were able to identify clinically relevant proteins associated with patients exhibiting HFpEF.

Fig. 7C shows the set of overlapping proteins that were identified using our NLP algorithm. Among these proteins, it is worth highlighting vinculin, desmin, and SPARC. Studies have linked the vinculin protein to cardiomyopathies [46–48]. At the same time, desmin and SPARC are associated with muscular structure and function [49], as well as fibrosis [50], which are biological processes relevant in the progression of HFpEF [51,52]. The cross-reference analysis with myocardial biopsies from HFpEF patients provides empirical validation of literature-based hypotheses. The results suggest that computationally identified high-ranked protein-disease associations are reflected in real world patient samples and may be relevant to the underlying disease pathophysiology of HFpEF.

### 3.7. Cross referencing high-ranked computational HFpEF proteins with clinical biopsies beyond the heart

In contrast to other CVDs and types of heart failure, HFpEF is often considered a systemic disease involving underlying pathologies outside the heart that lead to diastolic stiffness and dysfunction [53,54]. Accordingly, we conducted a cross-reference analysis of the top-20 ranked HFpEF proteins (the 20 proteins receiving the highest computational score within HFpEF) in its comorbidities and pathological mechanisms that we computationally queried from the PubMed database with clinical biopsies in other organs beyond the heart (extra cardiac) with protein expression datasets on from the Human Protein Atlas (Humap Protein Atlas version 24, October 2022) [55,56]. It is important to note that extracardiac samples used for protein expression analyses and cross-referencing were derived from healthy individuals rather than HFpEF patients. Therefore, conclusions regarding HFpEF as a systemic disease should be tempered, and the results should be interpreted as hypothesis-generating rather than confirmatory. However, with respect to protein localization, we anticipate that although expression levels may change in disease states, the overall tissue distribution of proteins is likely to remain largely unaltered.

**Fig. 8.**
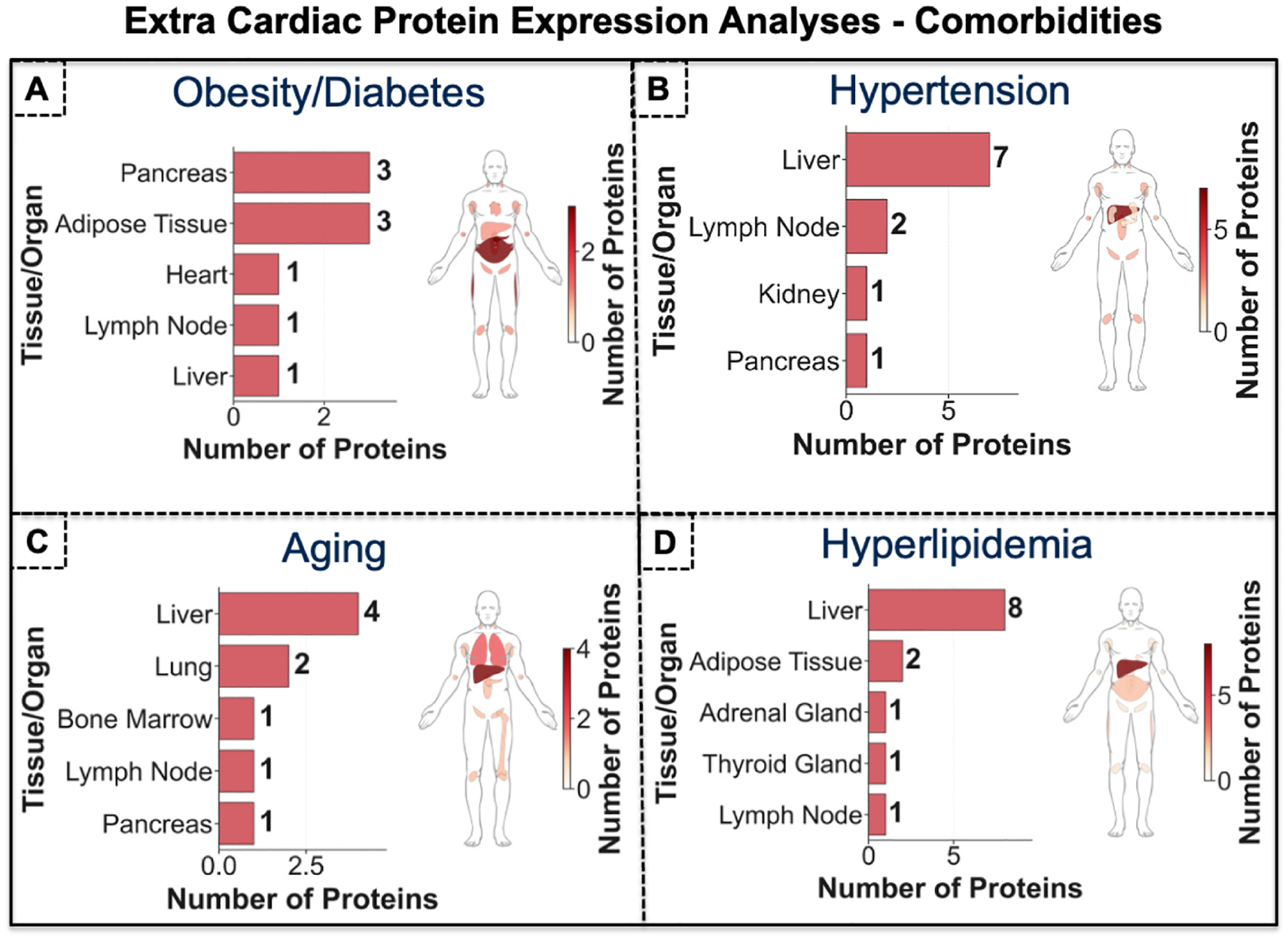
Cross-comparison of computationally prioritized HFpEF-associated proteins and comorbidities with extra-cardiac clinical biopsies. We conducted a cross-reference analysis of the computationally top-20 ranked HFpEF proteins. **(A)**, the top 20 HFpEF proteins shared with diabetes/obesity were mainly found in the pancreas and adipose tissue while only 1 protein was mainly found in cardiac biopsies. **(B)** The top 20 HFpEF proteins shared with Hypertension were mainly found in the liver (N = 7 proteins) and lymph nodes (N = 2 proteins) more than in cardiac biopsies (1 protein). **(C)** The top 20 HFpEF proteins in Aging were mainly found in the liver (N = 4) followed by lung biopsies (N = 2) and then bone marrow (N = 1), lymph nodes (N = 1), and pancreas (N = 1). **(D)** The top 20 HFpEF proteins in hyperlipidemia were found in liver biopsies (N = 8) and adipose tissue (N = 2) followed by lymph node (N = 1), adrenal gland (N = 1), and thyroid gland (N = 1). We found that the top ranked HFpEF proteins across its comorbidities that were computationally queried were mainly found in liver, pancreas and adipose tissue biopsies and not in the heart supporting the emerging opinion that HFpEF is a systemic disease.

As depicted in Fig. 8A, the top 20 HFpEF proteins shared with diabetes/obesity were mainly found in the pancreas and adipose tissue while only 1 protein was mainly found in cardiac biopsies. The top 20 HFpEF proteins shared with Hypertension (Fig. 8B) were mainly found in the liver (N = 7 proteins) and lymph nodes (N = 2 proteins) more than in cardiac biopsies (1 protein). The top 20 HFpEF proteins in Aging (Fig. 8C) were mainly found in the liver (N = 4) followed by lung biopsies (N = 2) and then bone marrow (N = 1), lymph nodes (N = 1), and pancreas (N = 1). Similarly, the top 20 HFpEF proteins in Hyperlipidemia (Fig. 8D) were found in liver biopsies (N = 8) and adipose tissue (N = 2) followed by lymph node (N = 1), adrenal gland (N = 1), and thyroid gland (N = 1). We found that the top ranked HFpEF proteins across its comorbidities that were computationally queried were mainly found in liver, pancreas and adipose tissue biopsies and not in the heart supporting the opinion that HFpEF is a systemic disease.

**Fig. 9.**
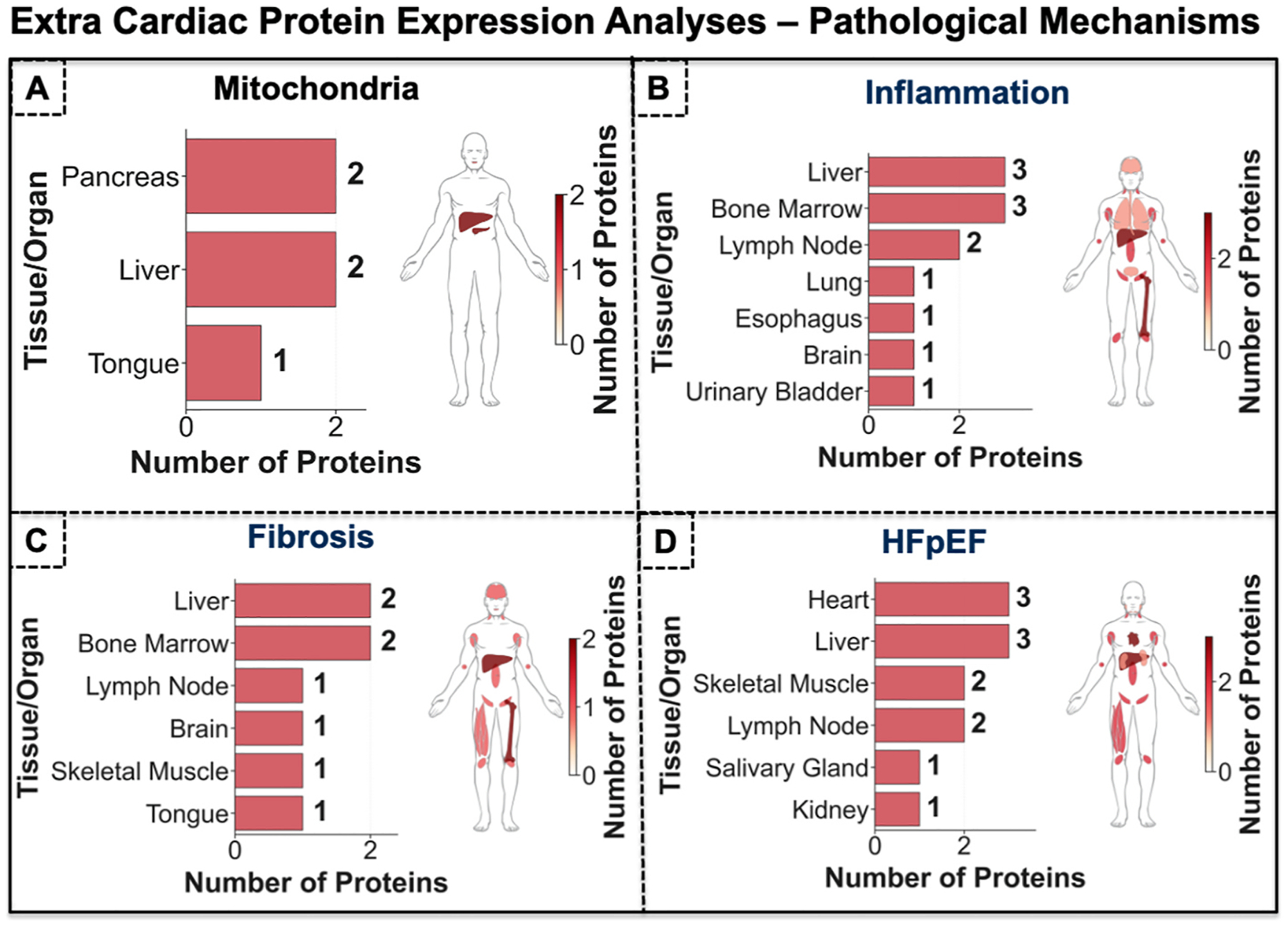
Cross-comparison of computationally prioritized HFpEF-associated proteins and pathological mechanisms with extra-cardiac clinical biopsies. **(A)** computationally ranked HFpEF proteins shared in mitochondria were mainly found in pancreas (N = 2) and liver (N = 2) biopsies. **(B)** The top 20 computationally ranked HFpEF proteins in inflammation were mainly found in liver, bone marrow and lymph node biopsies. **(C)** The top 20 computationally ranked HFpEF proteins in fibrosis were mainly found in bone marrow and liver biopsies. Lastly, the computationally top 20 ranked HFpEF proteins without taking comorbidities and pathological mechanisms into account (i.e., only focusing on HFpEF associations) were mainly found in liver, heart, brain, and lymph node biopsies. Overall, we observed that our list of computationally top ranked HFpEF proteins were mostly found in other organs than the heart implying that HFpEF has systemic pathophysiological biological processes rather than locally in the heart.

As depicted in Fig. 9A, computationally ranked HFpEF proteins shared in mitochondria were mainly found in pancreas (N = 2) and liver (N = 2) biopsies. Similarly, the top 20 computationally ranked HFpEF proteins in Inflammation (Fig. 9B) were mainly found in liver, bone marrow and lymph node biopsies. The top 20 computationally ranked HFpEF proteins in fibrosis were mainly found in Bone marrow and Liver biopsies (Fig. 9C). Lastly, the computationally top 20 ranked HFpEF proteins without taking comorbidities and pathological mechanisms into account were mainly found in liver, heart, brain, and lymph node biopsies. Overall, we observed that our list of computationally top ranked HFpEF proteins were mostly found in other organs than the heart implying that HFpEF has systemic pathophysiological biological processes rather than locally in the heart. It is important to note that the protein expression data for organ tissues in Human Protein Atlas version 24 are derived from healthy individuals and represent baseline physiological conditions, rather than from patients with HFpEF. Accordingly, our computationally derived protein data are compared against expression profiles from healthy individuals to assess the spatial distribution and tissue localization of these proteins throughout the body (Fig. 9). This also raises the possibility that protein spatial distributions can alter in disease states compared to baseline healthy states hereby affecting the heart during HFpEF [57].

## 4. Discussion

### 4.1. Central premise of this study

The central premise of this study is that a computational text-mining framework can systematically query, quantify, map, and prioritize protein–disease associations that are either shared or unique across six major cardiovascular disease (CVD) groups, followed by an in-depth HFpEF–focused analysis, using a corpus of over 38 million PubMed abstracts. Through this approach, we identified proteins associated with HFpEF, major comorbidities (aging, type 2 diabetes/obesity, hypertension, and hyperlipidemia), and key pathological mechanisms (mitochondrial dysfunction, inflammation, and fibrosis), and computationally quantified and prioritized these associations. This strategy provides a scalable means to generate pre-ranked molecular signatures that can inform and complement traditional omics-based laboratory research and clinical investigations. In this computational investigation, we present the first integrated framework to: (1) perform large-scale computational text mining centered on heart failure with preserved ejection fraction (HFpEF), incorporating multi-comorbidity and multi-pathophysiological protein mapping; (2) systematically cross-reference computationally identified proteins with human tissue-specific expression data derived from biopsy specimens; and (3) validate computational predictions using myocardial proteomic profiles from patients with HFpEF.

Looking forward, we aim to extend this framework beyond proteins to incorporate genes, transcriptomes, and metabolomes, enabling fully integrated computational multi-omics analyses across diverse diseases, risk factors, and additional domains of interest, including social determinants of health and lifestyle influences.

### 4.2. Unveiling molecular patterns and prioritizing protein-to-HFpEF associations across the PubMed database

To our knowledge, this study represents the first application of an NLP-based analytic framework to systematically evaluate protein–HFpEF associations across more than 38 million published studies. The resulting computational dataset, encompassing all proteins linked to HFpEF, its major comorbidities, and key pathological mechanisms, reflects how these proteins are reported and interconnected within the PubMed corpus, based on their frequency, distinctiveness, and contextual integrity across the domains of interest (as detailed in Supplemental Data 3). Among the HFpEF-related comorbidities, aging exhibited the largest number of associated proteins (11,628 proteins), followed by type 2 diabetes/obesity (9145 proteins). Proteins associated with aging also demonstrated the strongest linear regression relationship between their computational HFpEF scores and aging scores, indicating a greater number of highly ranked proteins, followed by type 2 diabetes/obesity and hypertension (Fig. 5E). Collectively, these findings suggest that aging and type 2 diabetes/obesity harbor a larger repertoire of proteins with strong HFpEF associations in the PubMed literature compared with hypertension and hyperlipidemia. Notably, our analysis included more than 3.7 million aging-related publications but only approximately 700,000 publications related to type 2 diabetes/obesity, suggesting that the protein associations in the latter may be concentrated within a smaller and more repetitive literature base.

In our assessment of major pathological mechanisms implicated in HFpEF, mitochondrial proteins demonstrated the highest linear regression correlation with HFpEF scores compared with proteins linked to inflammation and fibrosis (Fig. 6A–C). This pattern indicates that mitochondrial-related proteins include a greater proportion of high-scoring HFpEF-associated candidates, as further reflected in the violin plot (Fig. 6D). Remarkably, this enrichment occurred despite the mitochondrial literature being substantially smaller (approximately half the volume of inflammation-related publications and smaller than the fibrosis literature) yet still yielding the largest number of highly ranked HFpEF-associated proteins (Fig. 6D).

Lastly, we observed that the eight highest-scored HFpEF-associated proteins were consistently shared across comorbidities (Fig. 5F) and pathological mechanisms (Fig. 6E), whereas proteins with moderate to lower scores differed substantially between these two domains. The eight top-ranked proteins are summarized in Fig. 6F, including their common protein names, gene symbols, and primary biological functions. The convergence of these top eight proteins across comorbidities and pathological mechanisms (contrasted with the divergence observed among lower-ranked proteins) suggests that this subset of eight proteins represents key molecular drivers with convergent molecular networks underlying the pathogenesis of HFpEF.

### 4.3. Most computationally high-ranked proteins associated with HFpEF across the PubMed data base are predominantly expressed outside the heart

We aimed to validate our computational findings from across the whole PubMed data base of 38 million publications by cross-referencing our results with clinical myocardial biopsy proteomics data as well as data in other organs than the heart (extra cardiac). We observed that the top ranked HFpEF proteins across its comorbidities that were computationally queried (Fig. 8) were mainly found in liver, pancreas and adipose tissue biopsies and not in the heart. Similarly, the top ranked HFpEF proteins across fundamental pathological mechanisms (Fig. 9) were mainly found in pancreas, liver and lymph node biopsies and not the heart supporting the emerging proposition that HFpEF is a multiorgan systemic disease.

HFpEF is increasingly recognized as a systemic, multisystem disorder rather than a disease confined to the heart [58,59]. Accumulating evidence shows that HFpEF arises from the complex interplay of metabolic dysfunction, chronic inflammation, endothelial impairment, and multiorgan abnormalities involving adipose tissue, skeletal muscle, liver, kidneys, and the vasculature. These systemic perturbations drive myocardial remodeling and diastolic dysfunction, yet symptoms and disease severity often correlate more strongly with peripheral organ deficits than with cardiac structural changes alone [60–62]. This systemic perspective (supported by our computational findings) helps explain HFpEF’s strong association with comorbidities such as obesity, aging, type 2 diabetes, and hypertension, and provides a rationale for therapeutic strategies that target whole-body pathophysiology rather than focusing solely on the myocardium.

### 4.4. Text mining as a computational platform to quantify vast amounts of -omics data and synergize studies for biomarker evaluation and discovery

Although several proteins, such as plasma BNP and troponin-I, have been recognized as biomarkers with accurate diagnostic and gradation abilities in HF and IHD scenarios respectively, the ability for many other proteins to function as biomarkers in CVDs remains largely unexplored [63,64]. We demonstrate here, that a ML-empowered text mining approach can validate new biomarker candidates in CVDs by quantifying and connecting vast amounts of omics datasets from accumulated observations and scientific publications [65–68]. In this study, our text mining approach computationally queried, quantified, mapped, and prioritized exclusive proteomic molecular signatures between six main groups of CVDs. This approach can facilitate pre-selection of proteins (or any other multi-omics of interest) for targeted experimentation (targeted proteomic analyses) [69,70] and focused discovery rather than a shot-gun approach with long lists of proteins with unknown functions [71]. Our computational text mining application is able to connect accumulated (omics) data and unveil unseen patterns and molecular signatures in vast amounts of accumulated and disparate observations on the PubMed database [72–74].

### 4.5. Text mining as a powerful method to analyze electronic health records

With large patient textual datasets in the field of cardiology such as (i) data sets of demographic information in population studies, (ii) electrocardiogram measurements and adverse event reports, and (iii) echocardiographic imaging data, Computational Medicine has taken a pivotal role to facilitate clinical studies and precision medicine [16,66, 75–78]. NLP algorithms can facilitate the stratification and indexing of specific clinical events in large patient textual datasets of symptoms [79–81], side effects, and comorbidities from electronic health records [82–87], event reports, and reports from ECG and echocardiography. Accordingly, our further interest is to apply our text mining algorithms to patient notes in electronic health records and text reports from X-rays, echocardiography, ECGs, lab-test results, and CT/MRIs. Large-scale computational pipelines are a more feasible approach to uncover hidden patterns from vast amounts of patient textual datasets beyond the scope of proteins and effectively implies semantic relations between patient demographics and phenotypes [88–90].

### 4.6. From computational data science to bench work, to bedside

In the age of information technology and data science, modern medical research increasingly relies on the analysis of large biomedical (multi-omics) and patient datasets to enhance our understanding of complex heterogeneous human diseases [91–93]. In particular, the field of precision medicine aims to understand why therapies are effective in certain patients, while not effective in other patients with the same disease indicating complex heterogeneity in individuality [31]. Our text mining approach is able to uncover omics patterns (or any multi-omics of interest) in large text datasets to guide and synergize biomedical bench-work studies as well as clinical patient studies.

### 4.7. Limitations and literature bias

We acknowledge that the computational algorithms and derived protein scores are inherently constrained by multiple forms of literature bias. In each domain of interest, computational scoring is influenced by the volume of available publications, which varies substantially across domains. For example, the aging domain is characterized by the largest body of literature, followed by type 2 diabetes (T2D)/obesity and inflammation. One component of the protein scoring framework captures popularity, defined as the frequency with which a given protein is mentioned within a specific domain. Although scores are normalized across domains to enable inter-domain comparisons, disparities in corpus size can still introduce bias, as domains with larger literatures may disproportionately amplify protein mention counts.

Notwithstanding this potential bias, we observed that the mitochondria domain (despite having a comparatively smaller publication volume than aging, inflammation, or T2D/obesity) exhibited a total number of identified proteins that was of a similar magnitude, suggesting that corpus size alone does not fully account for protein representation. It may be argued that repeated mentions of well-studied proteins could skew rankings; however, from a methodological perspective, such repetition reflects biological and clinical prominence. Indeed, widely recognized biomarkers such as B-type natriuretic peptide (BNP) in heart failure or troponin I in ischemic heart disease appropriately achieve higher CaseOLAP composite scores due to their established relevance and extensive validation. In addition, literature co-occurrence bias refers to the inflation of apparent biological relationships caused by preferential co-mention of well-studied entities in the scientific literature, rather than by independent mechanistic or experimental evidence. It should be noted that all computational scores and correlations may be affected by literature co-occurrence bias.

Finally, it is important to emphasize that absence of evidence in the literature should not be interpreted as evidence of biological irrelevance. As our NLP-based approach relies on systematic mining of the PubMed database, it remains intrinsically subject to the prevailing biases of the biomedical literature, including differential research emphasis across domains.

## 5. Conclusions

We demonstrate that a combination of phrase-mining algorithms, and a large-scale network is effective in annotating large volumes of textual data to extract meaningful insights in a disease of interest. We computationally queried, quantified, mapped, and prioritized proteins exclusively attributed to 6 main CVDs with HFpEF as a deep dive study and unveiled underlying protein-to-disease correlations. Our computational framework can facilitate novel biomarker and drug target discoveries in any disease, risk factors or therapies of interest and complement clinical-, and preclinical omics studies, as well as text analysis in EHRs.

## Supplementary Material

1

2

3

## Appendix A. Supplementary data

Supplementary data to this article can be found online at https://doi.org/10.1016/j.compbiomed.2026.111599.

## Abbreviations and acronyms

NLP: natural language processing
CaseOLAP: context aware semantic online analytical processing
VITAL: ValIdated Text-mining using Advanced Language models
MeSH: medical subject headings
CVD: cardiovascular disease
CVA: cerebrovascular accident
IHD: ischemic heart disease
CM: cardiomyopathy
ARR: arrhythmia
VHD: valvular heart disease
CHD: congenital heart disease
HF: heart failure
HFpEF: heart failure with preserved ejection fraction
BNP: natriuretic peptide B
ACE: angiotensin converting enzyme
TNF: tumor necrosis factor
