# Supplementary material for: Natural language processing of biomedical text to map and prioritize protein–disease associations in HFpEF
Source: Comput Biol Med. Author manuscript; Available in PMC 2026 May 30. (PMC13221308; doi:10.1016/j.compbiomed.2026.111599)
Supplement: 3 [file NIHMS2179274-supplement-3.pdf]

Supplemental Data 3

Technical description of the CaseOLAP algorithm.

The Context-aware Semantic Online Analytical Processing (CaseOLAP) algorithm performs a phrase-mining analysis to facilitate phrase-based comparative summarizations of subsets of documents of interest. It begins with the construction of a functional document structure called a "Text Cube", with dimensions and cells informed by user provided document metadata. Each cell within the Text Cube corresponds to a subset of documents. A dimensional hierarchy is implemented to organize the Text Cube, providing each cell with a specific cell context (e.g., a parent cell, child cell, or sibling cell). The CaseOLAP algorithm uses three criteria to quantify the representation of each relevant phrase in the textual data: *integrity*, *distinctiveness, and popularity* (described later). The final CaseOLAP score for each relevant phrase is the product of these three parameters.

The CaseOLAP pipeline integrates several cutting-edge phrase-mining and machine learning algorithms. It has been compared with numerous existing algorithms, especially with their phrase segmentation enhancements, including TF-IDF+Seg, MCX+Seg, MCX, and SegPhrase and RepPhrase. The performance study results are shown in our study by Fangbo Tao et al.[1] RepPhrase was found to display greater precision than all the other alternatives.

In our study, we performed a phrase-mining analysis quantifying the associations of the 8,325 proteins to each of our six CVDs, and we utilized the workflow depicted in **Figure 1**. The textual data corpus of abstracts was first organized into a one-dimensional Text Cube, with one parent cell "Heart Diseases" and six child cells representing each CVD namely, IHD, CM, CVA, VD, ARR, and CHD.[2,3] As such, the dimensional hierarchy is relatively flat, as there is only one parent cell and six child cells that are siblings. Subsequently, the CaseOLAP algorithm was applied to rank the top-k phrases (the protein names and their synonyms listed in ***Supplemental Data 2***) based on the geometric mean of three scores: *integrity, distinctiveness*, and *popularity*. The three ranking criteria are described further: i) *integrity* (i.e., a good phrase describes an integral semantic unit that collectively refers to a meaningful concept); ii) *distinctiveness* (i.e., the relative relevance of a phrase in one subset of documents is distinguished from its relevance to other subsets of documents); and iii) *popularity* (i.e. a phrase with a higher popularity score appears more frequently in one subset of documents).

Quantitatively measuring these three concepts depends on the aforementioned cell context as well as the dimensional hierarchy. *Integrity* measures whether the phrase appears as a complete semantic unit describing a meaningful concept over multiple cells more frequently than expected by random chance. The *integrity* score denotes how often these representative phrases refer to the same concept by quantifying the number of times the words in each phrase appear together. In our case, as we provided a list of known protein names, the *integrity* score is the same for each of the 20,428 proteins. This is described further below. *Distinctiveness* is context-sensitive and subtle to define. It calculates the relevance of a protein name to a specific CVD by comparing the occurrence of the protein name in the target data set, i.e., the cell documents describing one CVD, to the contrastive data set, i.e., the cells of documents describing the remaining five CVDs.[1,4] *Popularity* indicates the prevalence of a phrase (protein name) within a target CVD. It depends on how frequently a protein name is mentioned within one CVD, and it is calculated only using the statistics from the cells of documents pertaining to that individual CVD. Rare protein names in a cell are ranked low, while an increase in their frequency of mention has a diminishing return. The mathematical equations denoting *integrity*, *distinctiveness*, and *popularity* are written below.

$$int\ (p,c) calculated\ by\ SegPhrase + \text{..................................}(1)$$

$$disti(p,c) = \frac{e^{rel(p,c)}}{1 + \sum_{c' \in K(p,c)} e^{rel(p,c')}} \text{..}(2)$$

$$pop\ (p,c) = \frac{\log(tf(p,c) + 1)}{\log cntP(c)} \text{...}(3)$$

Integrity, shown in equation (1), is directly derived from the corpus-wide SegPhrase+ computation [4] SegPhrase+ is a novel phrase-mining tool that utilizes the Random Forest algorithm to measure the quality of a phrase based on its popularity, discriminativeness, concordance, and completeness. First, a preprocessing step occurs to remove the noises or stop words from the textual data (e.g., words like “and”, “is”, “a”, “the”). Then, the Random Forest algorithm is used to extract the representative phrases from the document as complete semantic units. Furthermore, the Random Forest algorithm is used as a classification algorithm to judge the quality of a generated phrase. Then, a post-processing step identifies the best phrases from the output of the random forest algorithm. These steps are detailed in the research paper by Liu et al.[4]. In our

application of the CaseOLAP algorithm,[2,3] the representative phrases were the protein names (including abbreviations and synonyms), acquired from UniProt (uniprot.org). Thus, SegPhrase+ was not used to determine the *integrity* of these phrases, and the *integrity* score is the same across all 8,325 proteins. As the UniProt naming system is well established and broadly applied, the *integrity* score for each protein name is 1.0, the maximum score.

In the above equations (1 - 3), *p* refers to the phrase, or in this case, protein name, and *c* refers to a cell. Thus, *rel(p,c)* in the *distinctiveness* equation (2) represents the relevance of the phrase *p* to the cell *c*. *K(p,c)* in Equation (2) represents the collection of all cells neighboring cell *c* within the text cube, where cell *c* contains phrase *p*. Equation (2) calculates the probability (ranging from 0.0 to 1.0) of the *distinctiveness* of phrase *p* in cell *c* by taking the relevance score. Similarly, in the *popularity* equation (3), the term *tf(p,c)* represents the frequency of occurrence of phrase p in cell c, and *cnt(p,c*) is the total frequency count of all phrases in cell c. The equation of *popularity* calculates the ratio of log values of phrase frequency over total phrase frequency of all phrases in cell *c*, creating a range between 0.0 and 1.0 for all *popularity* scores. The three concepts viz: integrity $(int(p,c) \in [0,1])$, *popularity* $(pop(p,c) \in [0,1])$, and *distinctiveness* $(disti(p,c) \in [0,1])$ are independent and satisfy conjunctive conditions, and thus, the three quantities can be multiplied to obtain the final CaseOLAP score for each protein-disease pair as shown in equation (4) below.

$$r\ (p,c) = int(p,c) * pop(p,c) * disti(p,c) \ \ldots\ldots\ldots\ldots\ldots\ldots\ldots\ldots\ldots (4)$$

In the above equation, *r(p,c)* is the final CaseOLAP score for phrase *p* in document subset c.

1. Fangbo Tau QW, Taylor Cassidy, Lance R Kaplan, Clare R Voss, Jiawei Hang. Multi-Dimensional Phrase-Based Summerization in Text Cubes. *IEEE Data Engineering Bull*. 2016:74-84.
2. Liem DA, Murali S, Sigdel D, Shi Y, Wang X, Shen J, Choi H, Caufield JH, Wang W, Ping P, et al. Phrase mining of textual data to analyze extracellular matrix protein patterns across cardiovascular disease. *Am J Physiol Heart Circ Physiol*. 2018;315:H910-H924. doi: 10.1152/ajpheart.00175.2018
3. Sigdel D, Kyi V, Zhang A, Setty SP, Liem DA, Shi Y, Wang X, Shen J, Wang W, Han J, et al. Cloud-Based Phrase Mining and Analysis of User-Defined Phrase-Category Association in Biomedical Publications. *J Vis Exp*. 2019. doi: 10.3791/59108
4. Liu J, Shang J, Wang C, Ren X, Han J. Mining Quality Phrases from Massive Text Corpora. *Proc ACM SIGMOD Int Conf Manag Data*. 2015;2015:1729-1744. doi: 10.1145/2723372.2751523
